# Classification of Normal and Malicious Traffic Based on an Ensemble of Machine Learning for a Vehicle CAN-Network

**DOI:** 10.3390/s22239195

**Published:** 2022-11-26

**Authors:** Easa Alalwany, Imad Mahgoub

**Affiliations:** 1 Electrical Engineering & Computer Science, Florida Atlantic University, 777 Glades Road, Boca Raton, FL 33431, USA; 2 College of Computer Science and Engineering, Taibah University, Yanbu 46421, Saudi Arabia

**Keywords:** controller area network, ensemble learning, intrusion detection systems, machine learning

## Abstract

Connectivity and automation have expanded with the development of autonomous vehicle technology. One of several automotive serial protocols that can be used in a wide range of vehicles is the controller area network (CAN). The growing functionality and connectivity of modern vehicles make them more vulnerable to cyberattacks aimed at vehicular networks. The CAN bus protocol is vulnerable to numerous attacks, as it is lacking security mechanisms by design. It is crucial to design intrusion detection systems (IDS) with high accuracy to detect attacks on the CAN bus. In this paper, we design an effective machine learning-based IDS scheme for binary classification that utilizes eight supervised ML algorithms, along with ensemble classifiers. The scheme achieved a higher effectiveness score in detecting normal and abnormal activities when trained with normal and malicious CAN traffic datasets. Random Forest, Decision Tree, and Xtreme Gradient Boosting classifiers provided the most accurate results. Then we evaluated three ensemble methods, voting, stacking, and bagging, for this classification task. The ensemble classifiers achieved better accuracy than the individual models, since ensemble learning strategies have superior performance through a combination of multiple learning mechanisms. These mechanisms have a varied range of capabilities that improve the prediction reliability while lowering the possibility of classification errors. Our model outperformed the most recent study that used the same dataset, with an accuracy of 0.984.

## 1. Introduction

The future of the automotive industry will be controlled by intelligent connected vehicles (ICV). Crashes, congestion, and greenhouse gas emissions will be reduced as a result of advanced wireless technology that will allow vehicles to communicate with each other and others nearby in real-time [1]. Various research fields use vehicle nodes as communication messengers, such as vehicular ad hoc networks (VANET), the Internet of Vehicles (IoV), and communications from vehicles to everything else [2,3,4]. Vehicle communication protocols include controller area network (CAN), FlexRay, and MOST (Media-Oriented Systems Transport), all of which are well-established standards.

The wide utilization of the CAN bus in vehicles is done for a diverse range of reasons, among which are the reduction of costs, the improvement of real-time communication, and the simplicity of installation. Broadcast data transfer, priority arbitration, and lower latency are characteristics of the CAN bus. However, these particular characteristics are the cause of its vulnerabilities [5,6,7]. Traditional security mechanisms, such as network segmentation, encryption, and authentication, are incompatible with in-vehicle networks because of CANs’ lack of support and the possible violation of timing constraints that can occur in CAN communications [8,9]. Therefore, vehicular networks’ openness to malicious threats puts the modern IoV in danger of malicious messages. Some vehicle functions, including acceleration, steering, and braking, might be controlled by an attacker. In [10], a Jeep Cherokee was hacked, and its functions were controlled. Attacks on vehicles endanger the lives of drivers, passengers, vulnerable pedestrians, and other vehicles.

Intrusion detection systems (IDSs) have become a proposed solution to detecting attacks [11]. The primary goal of any IDS is to identify network traffic patterns that may indicate suspicious activity. Researchers have found the integration of machine learning (ML) models into IDSs to detect attacks in IoV networks highly effective [12,13]. This research is being conducted using ML classifiers that have been trained to predict normal and malicious traffic using prebuilt datasets. Security solutions broadly employ classification algorithms which have proven to be extremely powerful in threat and attack detection and prevention. If the design of in-vehicle network intrusion detection includes classification algorithms, it is possible to learn how normal network traffic works and to identify when CAN buses are acting abnormally [14,15,16].

The CAN bus protocol is vulnerable to numerous attacks, as it is lacking security mechanisms by design. It is crucial to design IDSs with high accuracy to detect attacks on the CAN bus. Refs. [17,18,19] proved that the use of ensemble methods can enhance accuracy in intrusion detection more than a single classifier. Ensemble learning aims to achieve better classification results through the use of different classifiers that are combined into a single classifier. The strength of the ensemble learning strategy is that it allows learning mechanisms with varying capabilities to support each other, resulting in a higher prediction reliability and a low error rate.

In this study, we propose a solution that aims to build an effective IDS framework by using well-tuned supervised ML models and ensemble learning methods. We designed an effective machine learning-based IDS scheme for binary classification that utilizes eight supervised ML algorithms, along with ensemble classifiers. We combine all supervised models using three ensemble methods: voting, stacking, and bagging. The advantage of the ensemble learning strategy is that it enables learning mechanisms with different capabilities to support one another for this classification task. Our main contributions are illustrated as follows:

- We build and evaluate eight supervised models with hyper-parameter tuning and data balancing. These models included a Random Forest (RF), Decision Tree (DT), and Xtreme Gradient Boosting Classifier (XGBoost). We use three ensemble methods to combine the eight supervised ML models to increase the attack detection performance of our scheme. We carry out the performance evaluation in terms of accuracy, precision, recall, F1-score, and area under curve receiver operator characteristic (ROC-AUC).
- We provide a comparison of our models with recently proposed models that utilized the same dataset. Our models achieved higher attack detection performance.

The remainder of this study is organized as follows. Section 2 shows the related work. Section 3 presents the CAN background and its attack types. Section 4 demonstrates the proposed models. Section 5 presents the results and discussion. Finally, Section 6 concludes the paper.

## 2. Related Work

CAN-based in-vehicle network attack detection has been the focus of numerous studies because researchers have deemed IoV IDS research critical in recent years. Discoveries made by earlier research have given hints of the possibility that attackers could inject bus messages into CAN after exploiting discovered attack surfaces, which are categorized as wireless and physical [20]. During earlier research, these points of entry for attacks into the CAN bus of a target vehicle were identified. A malicious node can use these points of entry.

Refs. [6,21] are among the recent studies that offer categorization and expansive CAN bus vulnerability and attack reviews. Their body of work also presents solutions to the reviewed vulnerabilities and attacks. According to these two research papers, eavesdropping is possible when traffic is not encrypted; protocol misuse led to denial of service (DoS) and replay attacks; data insertion attacks are possible when messages do not have authentication. These are the three significant vulnerabilities and associated attacks. In [22], an approach is proposed for in-vehicle networks’ CAN intrusions to identify intrusion classification models using the CAN-Intrusion-Dataset. The authors implemented K-Nearest Neighbor (KNN) and Support Vector Machine (SVM) algorithms in their object classification.

The supervised machine learning classifiers RF, KNN, and XGBoost are compared in classifying attack and non-attack messages using the CAN-Intrusion-Dataset in [23]. Compared with KNN for intrusion detection, RF and XGBoost showed better precision, recall, and F-score values. However, an unbalanced dataset was used, and further improvements are needed.

The authors of [24] have proposed ensemble learning methods combined with Decision Trees to classify normal and malicious traffic. The detection rate for both the Decision Tree and bagging was 0.97. However, using AdaBoost, Naïve Bayes, Logistic Regression, and a Neural Network (NN) has not shown further improvement. Moreover, the model is trained with the default parameters.

The authors of [25] have proposed Deep Convolutional Neural Network (DCNN) and SVM models for the detection of normal and malicious traffic. The performances of the detection models are improved using the hint data that are proposed. The detection performances of the models for attack types known via hints improved when using hint data to train the model. Further improvements are needed to reach the accuracy required for use in the real world, since the models used in the paper had a maximum accuracy of 0.95.

In [26], optimal performance is achieved using traditional ML algorithms. The DT algorithm achieved the highest accuracy of itself, SVM, KNN, and RF. The DT algorithm scored 0.9532 in the correct classification of individual scenarios as normal or attack. However, the model’s detection performance needs to be improved further.

In this study, we propose a solution that aims to build an effective IDS framework by using well-tuned supervised ML models and ensemble learning approaches. Table 1 shows our scheme compared to recently proposed approaches in terms of ensemble learning, data balancing, and hyperparameter tuning.

In Table 1, "+" means the method is used and is considered as a strength, "—: means the method is not used and is considered as a weakness.

## 3. Background and Attack Types

### 3.1. Overview of CAN Bus

The CAN was introduced for vehicles in 1986 by Robert Bosh GmbH [27]. The actuator, sensor, controller, and other nodes from real-time applications are the main components that communicate with one another [28]. The CAN bus protocol involves the use of a broadcast mechanism that sends data/messages to all nodes of the network. Multiple CAN buses can be found in modern vehicles. Each bus can perform a variety of functions, such as engine control and brake control [29]. One of the primary benefits of the CAN system is the ability to add nodes without requiring complex programming. This has facilitated the process of integrating external systems with it, and it will continue to function normally, as before integration. Another benefit of the CAN system is fault detection that can be done with extreme precision. It also has a fault confinement feature that allows it to shut down automatically if severe errors occur. If one node fails, the others will continue to function normally [30].

**Figure 1:**
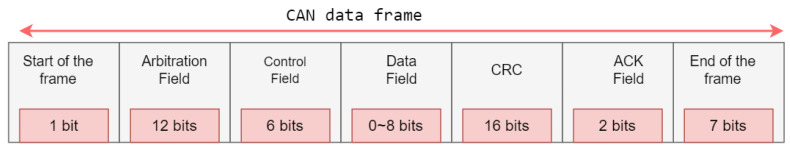
The structure of the CAN bus frame.

The transmission of CAN packets or messages usually takes place over the CAN bus data frame. The ranking of different CAN packets by their importance gives the data frame the highest scores amongst all the available CAN packets. The transmission of user data takes place in the data field [16]. The CAN packet structure is made up of seven fields: start of the frame, arbitration field, control field, data field, cyclic redundancy check field, acknowledge field, and end of the frame. Figure 1 illustrates the structure of the CAN bus frame.

*Start of The Frame (1 bit):* It utilizes one bit to inform all nodes about the beginning of CAN message transmission.

*Arbitration Field (12 bits):* It is known as CAN ID; it identifies the priority of received message. A lower value for the CAN ID message means it has high priority.

*Control Field (6 bits):* It includes Data Length Code (DLC), which indicates the byte length of the data field.

*Data Field (0 to 8 bits):* It is responsible for actual data transmission.

*Cyclic Redundancy Check (CRC) Field(16 bits):* It is used to detect the validity of packets.

*Acknowledge (ACK) Field (2 bits):* It confirms that the CAN packets are received successfully by the receiver nodes.

*End of The Frame (7 bits):* It indicates that the CAN message has been completed.

### 3.2. CAN Bus Attacks

The lack of suitable security support in the CAN protocol itself resulted in a limitation on the mechanisms to secure the communications between vehicles. Researchers have extensively investigated the security limitations of the CAN bus in real vehicles and in laboratories. Cost reduction, enhancing real-time communication, and installation simplification were among the reasons for using the in-vehicle CAN bus. The use of the CAN bus in vehicles introduces several security vulnerabilities [7,9,16]. The three characteristics of the CAN bus that cause vulnerabilities are broadcast data transfer, priority arbitration, and low latency. Consequently, traditional security mechanisms, including cryptography techniques, authentication, and integrity, are incompatible with in-vehicle networks because the CAN bus does not support them [31,32]. Bus injection and CAN bus DoS attacks have become increasingly common [33]. Attacks in various environments show that attackers can take control of various vehicle parts, such as the brakes and steering [7]. The points of entry attacks into the CAN bus can be wireless or physical. With more connections and interfaces to the outside, the CAN bus network becomes easier to hack [15]. The main type of inter-vehicle attack is a message injection attack, such as a DoS, spoofing, replay, or fuzzing attack [11]. *Flooding (DoS):* This involves congesting the network bus by sending the CAN bus segment multitudes of traffic messages, making it difficult for legitimate service traffic to go through the bus because it will always be fully utilized. The CAN bus’s lack of authentication mechanisms makes DoS and message poisoning attacks prevalent and unmitigated. Arbitration schemes such as message-ID-priority schemes help mitigate DOS attacks on other connected ECUs. *Spoofing:* Spoofing of the source-destination enables the attacker to inject into the CAN bus messages which can enable the attacker to take control of a specific set of the attacker’s desired functions. The broadcast mechanism the CAN bus employs makes it possible for any of the bus-connected electronic control units (ECUs) to sniff CAN frames because there is no encryption mechanism for the data traffic on the CAN bus. *Replay:* This type of attack involves replaying or injecting previously extracted normal traffic into the compromised CAN bus at a specific time. This attack is possible because of the CAN frames’ lack of authentication and integrity. *Fuzzing:* The attacker makes a vehicle’s behavior unexpected due to the injection of random messages.

## 4. Materials and Methods

### 4.1. Supervised ML

We propose binary classification of normal and abnormal traffic for CAN bus messages. This proposed solution aims to build an effective IDS framework by using supervised ML models. We used supervised ML models. These models were Random Forest, Decision Tree, Gaussian Naïve Bayes, Logistic Regression, AdaBoost, K-Nearest Neighbor, and Gradient Boosting. The models were evaluated by using well-known evaluation metrics. These metrics were accuracy, precision, recall, F1-score, and ROC.

#### 4.1.1. Dataset Description

We used the CAN intrusion dataset provided by The IEEE DataPort [34].The dataset is chosen because it has been recently collected in 2020. Moreover, the dataset is completely labeled, which is needed to support our proposed supervised learning models. The authors of the dataset collected have millions of CAN bus messages from the real data of a vehicle, and then cybersecurity attacks are launched. The features utilized in the dataset are described in Table 2. Table 3 depicts the distribution of malicious messages based on the type of attack and the number of normal messages sent.

#### 4.1.2. Workflow of Supervised Models

**Figure 2:**
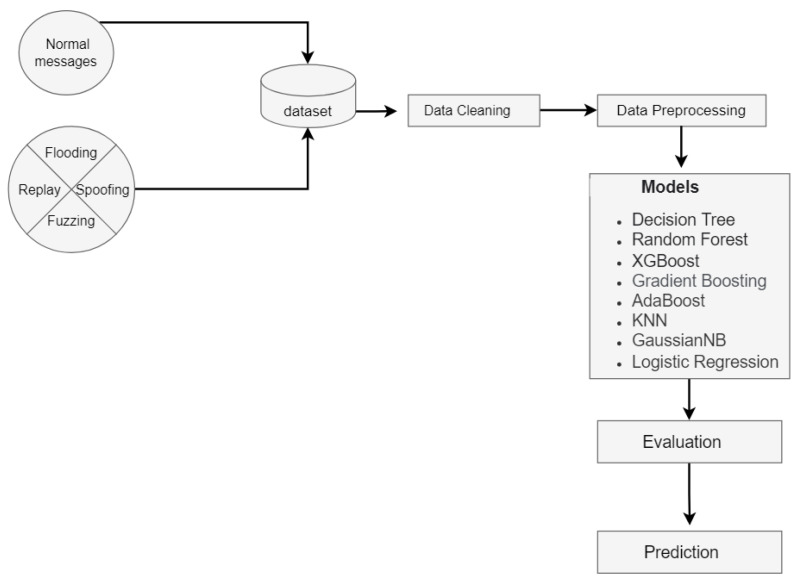
The overview of the workflow with the supervised models.

The workflow is illustrated in Figure 2. First, we merged the malicious and benign messages into a single dataset. The data were then cleaned and preprocessed, including oversampling for data balancing. Then, a variety of ML classification models were utilized. Finally, the classification models were evaluated by using well-known evaluation metrics.

#### 4.1.3. Environment Tools

The development environment for this scheme was a Jupyter Notebook. This is one of the applications offered by Anaconda. The scheme was implemented using the Python programming language. Python was chosen because of its effectiveness, scalability, and stability; it also offers a variety of evaluation metrics, which were useful for this work.

#### 4.1.4. Data Cleaning

Since the raw dataset has not processed, several methods were used, such as cleaning, removing duplicates, and removing null values. The mean, median method was used for filling null values.

#### 4.1.5. Data Preprocessing

Data mining utilizes this technique to turn unstructured data into an understandable format. In the real world, data are frequently incomplete and mismatched. There are many different approaches to preprocessing the data that can be used. During the preprocessing phase of the data, label encoding was utilized. Label encoding is the process of assigning appropriate integer values to each element of a dataset. This procedure involves preparing the data so they can be used by models. Specifically, the data are separated into training and test sets. With this method, 80% of the data were used as the training set and 20% as the test set. We utilized oversampling. Oversampling can help fix the class unbalance issue of data. Attack samples usually make up a considerably smaller percentage compared to normal samples for network data obtained from the real world, so real-life scenarios yield low detection rates and biased models. Using resampling methods such as synthetic minority oversampling techniques (SMOTE) and random sampling, on the other hand, can solve the problems of class unbalance by creating minority classes in some new cases and balancing the dataset [35]. Increasing the classification accuracy of the models is the primary goal of using balanced data for model training to improve performance [36]. In [37,38], oversampling was introduced into IDS. Oversampling is a useful technique for enhancing the performance of a classifier [39].

#### 4.1.6. Classification Models

Using the dataset, eight effective classifiers were utilized to predict malicious and normal messages. We used the most popular single ML models: Logistic Regression (LR), Gaussian Naive Bayes (GaussianNB), k-NN, RF, Gradient Boosting, AdaBoost, DT, and XGBoost. We used timestamp, arbitration-ID, DLC, and data as inputs; and the target of the output was to predict malicious and normal messages, where 0 represents normal and 1 represents malicious.

### 4.2. Ensemble Classifiers’ Learning

We proposed an effective intrusion detection system that is based on machine learning and uses classification-algorithm-based ensemble classifiers to improve the IDS detection ability. We choose three ensemble methods, stacking, bagging, and voting, because predictions are weighted according to the significance of the single classifiers and combined to generate the sum of the weighted probabilities. Vehicle networks receive a significant boost in the protection of their safety with ML classification. The performance demonstrated by single-model learning is considerably inferior to that of ensemble learning. Ensemble learning strategies’ superior performance compared to the single learning results. The combination of multiple learning mechanisms, which have a varied range of capabilities, improves prediction reliability while lowering classification errors. Ensemble learning is more effective at improving the classification accuracy of the entire system. We used the following ensemble methods.

Stacking ensemble learning is a common way to combine the results of multiple basic classifiers through a meta-model. It attempts to improve the ensemble’s performance by fixing errors. The main idea is to apply a different classifier to fix the weaknesses in the prior classifiers [40]. Stacking consists of two phases. In the first phase, there are eight-model base learners (Gradient Boosting, GaussianNB, k-NN, LR, RF, AdaBoost, DT, and the XGBoost classifier). The second phase consists of a meta-learner with one model (Logistic Regression). Stacking uses these two phases to identify how to efficiently combine the single models in the base learners with the meta-learner.

A voting classifier is a model that can select the most desirable option from its ML models using a voting mechanism. The types of voting are soft and hard voting. We used soft voting because it gives the highest weights to votes with the most confidence, considering how important each classifier is in the final decision [41]. After aggregating the results of each classifier, voting is utilized to predict the final result based on the maximum vote majority. We used eight supervised models and let the voting ensemble predict the final one.

**Figure 3:**
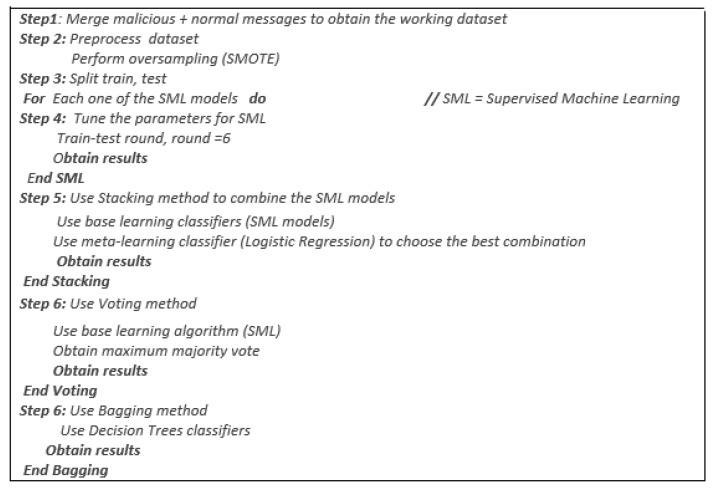
Our proposed IDS scheme development and evaluation steps.

Bagging has bags of similar or dissimilar types of base classifiers. The classifier helps to minimize the variance of the base classifier to increase the performance. It essentially aims to improve the precision and stability of supervised models [42,43]. The purpose of the ensembles classifiers is to improve the performance of individual ML models. We used the bagging classifier with the Decision Tree model to minimize variation and prevent overfitting. Figure 3 shows our proposed IDS scheme development and evaluation steps.

#### Workflow of Ensemble Classifiers

**Figure 4:**
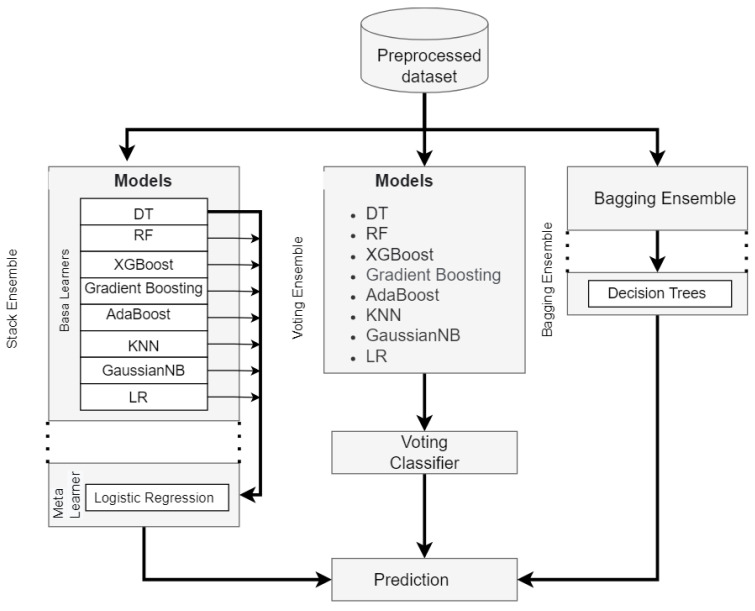
The overview of workflow of ensemble classifiers.

We used the supervised models’ preprocessed dataset (Section 4.1). As seen in Figure 4, we also implemented three ensemble classifiers: voting, bagging, and stacking. Accuracy can be improved through implementing processes that enable the working together of multiple ML algorithms on a single ensemble classifier prediction. The single-ensemble-platform integration of a diversified set of the classifiers used in (Section 4.1) can help enhance the system’s overall classification accuracy.

## 5. Results

### 5.1. Metrics for Evaluation

To evaluate our scheme, we used the evaluation metrics of accuracy, recall, precision, receiver operator characteristic (ROC), and F1-score, as defined below in terms of true positives (*TP*), false positives (*FP*), true negatives (*TN*), and false negatives (*FN*).

*Accuracy* is defined as the ratio of correctly predicted samples to total samples:

(1) $$Accuracy=\frac{TP+TN}{TP+TN+FP+FN}$$

*Precision* is defined as the ratio of correctly identified positive samples (*TP*) over the total number of accurately and erroneously classified positive samples.

(2) $$Precision=\frac{TP}{TP+FP}$$

*Recall* is defined as the ratio of the number of positively identified samples (*TP*) to the total number of samples predicted to be positive.

(3) $$Recall=\frac{TP}{TP+FN}$$

*F1-score:* The range of the F-1 score, which enables the computing of precision and the recall harmonic mean, is usually between 1.0 and 0.0. A higher value of the F-1 score indicates a higher degree of perfectness in precision and recall.

(4) $$F1=\frac{2*Precision*Recall}{Precision+Recall}=\frac{2*TP}{2*TP+FP+FN}$$

*Receiver Operator Characteristic:* ROC AUC is one of the most important indicators. It shows where to attack, and normal-scenario classifications were made more accurately when the proposed model was used. A high ROC value indicates an effective classification model. Specificity (true positive rate, or TPR) and sensitivity (true negative rate, or TNR) are also taken into account; sensitivity is equal to recall.

(5) $$Specificity=\frac{TN}{FP+TN}$$

### 5.2. Results and Discussion

We aimed to create a balanced dataset in order to avoid any potential bias issues that could affect the accuracy of the classification models. We oversampled the dataset to have an equal number of malicious and benign messages. We performed tuning for the models. Model tuning is responsible for improving the performance of a ML algorithm. Hyperparameter tuning involves special values or weights that affect the learning process of an algorithm in order to improve prediction accuracy. We used the random search for the optimal set of hyperparameters. The hyperparameters of the models that were employed are shown in Table 4. Each model was evaluated and assessed based on accuracy, precision, recall, F1-score, and ROC. The proposed solution aims to build a more effective IDS framework by using well-tuned and balanced supervised ML models.

**Figure 5:**
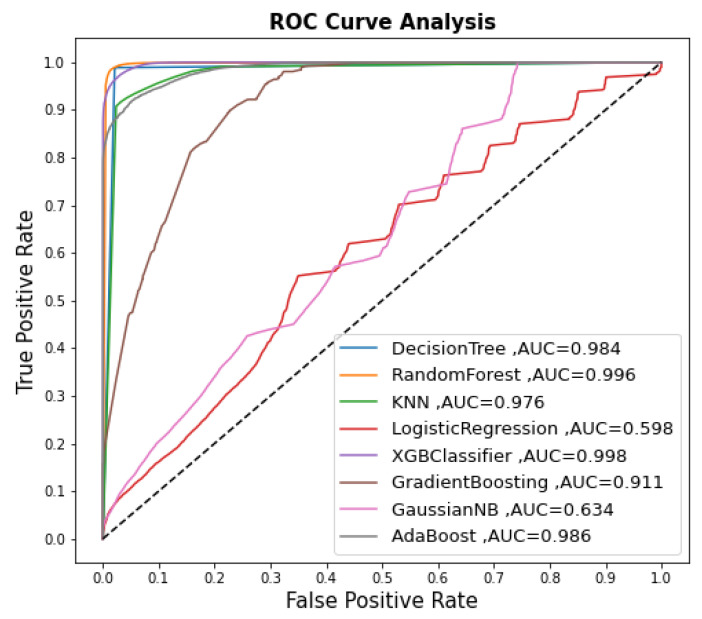
The ROC results for supervised models.

As shown in Table 5, the RF and DT models had the highest accuracy of 98.3%, followed by the XGBoost model, which had an accuracy of 97.0%. The GaussianNB and LR models were the least accurate, at 57.3% and 56.8%, respectively. KNN, AdaBoost, and Gradient Boost provided good accuracy: 93.4%, 93.3% and 82.6%, respectively. After applying correlation feature selection, we found that the majority of input features had a low correlation with the target variable. The Logistic Regression model achieved low accuracy. On the other hand, the Random Forest model achieved high accuracy because it has its own feature selection. Figure 5 shows the ROC results of the developed supervised models.

**Figure 6:**
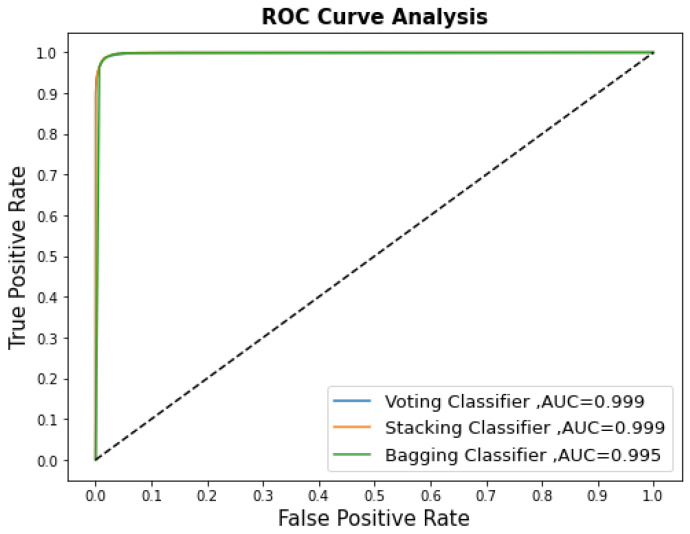
The ROC results for the ensemble classifier models.

The main aim of this research was to design an effective machine learning-based IDS scheme for binary classification that utilizes eight supervised ML models, along with ensemble classifiers. The developed supervised ML models were Gradient Boosting, GaussianNB, k-NN, LR, RF, AdaBoost, DT, and XGBoost. These models were fine-tuned and data-balanced. The most accurate models were the Random Forest, Decision Tree, and Xtreme Gradient Boosting Classifier. The ensemble learning strategy is superior because it allows learning mechanisms with varying capabilities to support each other. Table 6 shows a summary of the quantitative performances of the three ensemble classifier models. These were stacking, bagging, and voting classifiers. Compared to the single models, the proposed ensemble classifier models improved the performance. Stacking showed effective performance through scores of 0.984 for accuracy, 0.980 for precision, 0.989 for recall, and 0.984 for F1. Stacking is a two-stage procedure. In the first stage, it has eight model-based learners (Gradient Boosting, GaussianNB, k-NN, LR, RF, AdaBoost, DT, and the XGBoost classifier). The second stage is a meta-learner with one model (Logistic Regression). Stacking utilizes these two stages to learn and discover how to best combine the single models in the base learners with the meta learners. When compared to bagging, stacking showed high scores for precision, recall, and ROC. When compared to voting, stacking showed a high score for recall. The ROC curve was plotted, and the area under the curve (AUC) was calculated to evaluate the intrusion detection performance. The ROC is a graph that compares the true positive rate to the false positive rate at varying thresholds and is used to illustrate a binary classifier’s ability to differentiate between two classes (normal and malicious messages). Figure 6 depicts a high ROC value, which indicates that the ensemble classifiers are effective at solving the CAN bus intrusion detection problem.

**Figure 7:**
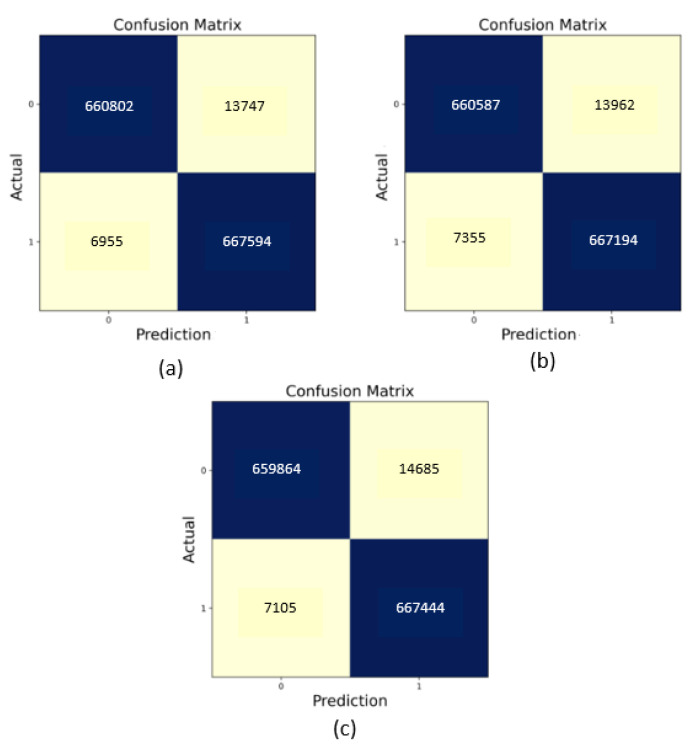
(**a**) The confusion matrix of the stacking. (**b**) The confusion matrix of the voting. (**c**) The confusion matrix of the bagging.

Figure 7 illustrates the confusion metrics of the three ensemble learning models. In the proposed system, the confusion metrics of TP, FP, TN, and FN are important to evaluate and classify CAN messages as normal or attack messages.

**Figure 8:**
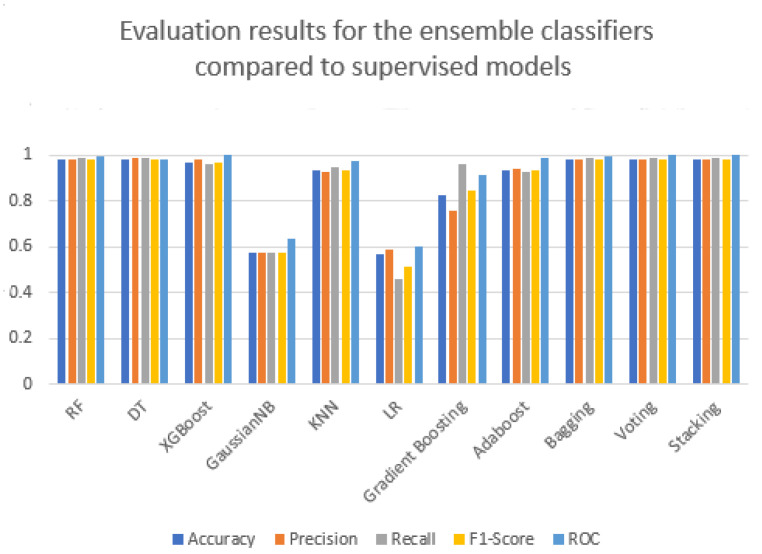
The performance of ensemble classifiers compared to supervised models.

As seen in Figure 8, compared to the eight supervised models, the three ensemble learning strategies performed better. In terms of the five metrics, the Gaussian Naive Bayes and Logistic Regression models did not achieve acceptable results in the supervised models or ensemble learning. However, stacking outperformed both ensemble learning and supervised models.

The stacking and voting methods’ run-times were in the tens of seconds; however, it was well worth it in order to achieve better accuracy. Stacking is a method that needs two different training phases, consisting of nine training processes, eight base classifiers, and a final classifier on the meta-learner. Voting used the eight supervised models to predict the result. However, bagging took less time, since it used the Decision Tree model. Consequently, time performance is dependent on computing power.

Table 7 displays the proposed method compared to recently proposed, promising approaches in [24,25,26]. In terms of evaluation metrics for the same dataset, the performance of our model is encouraging. Consequently, the experimental results show that the proposed IDS can successfully detect benign and malicious network traffic data and identify attacks on in-vehicle networks. IDS-based ensemble methods enhance the performance of the detection of malicious network traffic. The ensemble classifiers were developed using the bagging, voting, and stacking models. Evaluation results showed that the proposed ensemble models achieved the highest binary classification performance.

## 6. Conclusions

In this paper, we proposed an ML-based IDS scheme for detecting intrusion attacks on the CAN bus with the goal of improving attack detection accuracy. We developed and tested the results of eight supervised machine learning models—Random Forest, Decision Tree, Gaussian Naïve Bayes, Logistic Regression, AdaBoost, K-Nearest Neighbor, and Gradient Boosting—in identifying normal and malicious CAN traffic messages. The results of these models showed that the Random Forest, Decision Tree, and Xtreme Gradient Boosting classifiers are the most accurate. Then, we combined all supervised models using three ensemble methods: voting, stacking, and bagging. The advantage of the ensemble learning strategy is that it enables learning mechanisms with different capabilities to support one another for this classification task. The ensemble classifiers outperformed the supervised classifiers and improved the effectiveness of the supervised ML models. However, the ensemble classifiers required longer run-times, particularly Stacking and voting—in the tens of seconds. Compared to bagging, Stacking showed higher precision, recall, and ROC scores. Compared to voting, Stacking showed a higher recall score. The stacking model had an accuracy score of 0.984, a precision score of 0.980, a recall score of 0.989, an F1-score of 0.984, and an ROC score of 0.99. These are noticeable improvements over the other models. In addition, stacking outperformed the models in [24,25,26] in terms of accuracy, precision, and F1-score, as shown in Table 7. In our future work, we will develop a multi-classification scheme to detect and classify attacks on the CAN bus system.

## Figures and Tables

**Table 1:** Comparison of our scheme with recently proposed models.

Citation	Year	Machine Learning Models	Data Balancing	Hyperparameter Tuning	Ensemble Classifiers
[24]	2020	+	—	—	+ (Bagging)
[25]	2021	+	Not Reported	—	—
[26]	2021	+	Not Reported	+	—
Ours	2022	+	+	+	+ (Stacking, Voting, and Bagging)

**Table 2:** The features and descriptions of the dataset.

Features	Descriptions
Timestamp	Record time
Arbitration-ID	CAN message identifier
DLC	Data Length Code
Data	CAN data field
Target	Determine the type of message (normal and malicious)

**Table 3:** The distribution of malicious messages and the normal messages.

Message Type	Count	Percentage (%)
Normal	3,372,743	91.846523
Flooding	154,180	4.198629
Fuzzing	89,879	2.447585
Replay	47,593	1.296052
Spoofing	7756	0.211211
TOTAL	3,672,151	100

**Table 4:** Hyperparameter tunings for the supervised models.

Algorithm	Hyperparameter
RF	Estimators = 20
DT	Criterion = ’entropy’, Splitter = ’best’
KNN	Metric = ’minkowski’, Weights = ’uniform’
Gradient Boosting	Eestimators = 20, Maximum Depth = 3
Ada Boost	Estimators = 1000

**Table 5:** Evaluation results for the supervised models.

Models	Accuracy	Precision	Recall	F1-Score	ROC
RF	0.983	0.979	0.988	0.984	0.996
DT	0.983	0.987	0.988	0.983	0.983
XGBoost	0.970	0.980	0.960	0.970	0.998
GaussianNB	0.573	0.573	0.574	0.573	0.644
KNN	0.934	0.926	0.944	0.935	0.976
LR	0.568	0.588	0.458	0.515	0.598
Gradient Boosting	0.826	0.756	0.964	0.847	0.911
Adaboost	0.933	0.941	0.924	0.932	0.986

**Table 6:** Evaluation results for the ensemble classifiers.

Models	Accuracy	Precision	Recall	F1-Score	ROC
Bagging	0.984	0.979	0.988	0.984	0.995
Stacking	0.984	0.980	0.989	0.984	0.999
Voting	0.984	0.980	0.988	0.984	0.999

**Table 7:** Our proposed method compared to recently proposed promising approaches that used the same dataset.

Citation	Year	Models	Accuracy	Precision	Recall	F1-Score
[24]	2020	Random Forest	0.97	0.90	0.99	0.94
		Bagging	0.97	0.90	0.99	0.94
		Naïve Bayes	0.96	0.90	0.94	0.92
		Ada Boosting	0.81	0.41	0.53	0.46
		Logistic Regression	0.96	0.79	0.76	0.77
		Neural network	0.96	0.78	0.78	0.78
[25]	2021	DCNN	0.9537	0.9671	0.9262	0.9451
		SVM	0.8448	0.9571	0.6545	0.7517
[26]	2021	Decision Tree	0.9532	0.9463	0.9558	0.9608
		KNN	0.9248	0.9141	0.9390	0.9215
		Random Forest	0.9448	0.9448	0.9245	0.9216
		SVM	0.9440	0.8854	0.9542	0.9512
Ours	2022	Voting classifier	0.9845	0.9804	0.9887	0.9846
		Stacking	0.9847	0.9804	0.9891	0.9847
		Bagging	0.9840	0.9793	0.9889	0.9841

## Data Availability

Data available upon request.
